# A Case of Acquired Factor V Inhibitor Following Nivolumab Administration

**DOI:** 10.7759/cureus.21670

**Published:** 2022-01-27

**Authors:** Wataru Kida, Muneo Nakaya, Akiko Ito, Yasuji Kozai, Masato Bingo

**Affiliations:** 1 Department of Otolaryngology–Head and Neck Surgery, Tokyo Metropolitan Tama Medical Center, Tokyo, JPN; 2 Department of Hematology, Tokyo Metropolitan Tama Medical Center, Tokyo, JPN; 3 Laboratory Medicine, Tokyo Medical University, Tokyo, JPN

**Keywords:** chemotherapy, hypopharyngeal, pd-1 inhibition, nivolumab, acquired factor v inhibitor

## Abstract

Acquired factor V inhibitor (AFVI) is a very rare disease. We presented herein a case of hypopharyngeal cancer in which AFVI developed after nivolumab administration. Blood test findings two weeks after the first dose of nivolumab showed a significant prolongation of prothrombin time (PT) and activated partial thromboplastin time (APTT), indicating a marked abnormality in the coagulation function.

Factor V activity had decreased significantly and was below the detection limit (<3%), and the factor V inhibitor level was as high as 16 Bethesda units (BU)/mL. His underlying illness was a malignant tumor, but we considered that nivolumab administration was the cause of AFVI, considering the time when coagulation abnormality developed. No significant bleeding tendency was observed in the subsequent course, and the AFVI was followed up without treatment. To the best of our knowledge, the present study is the first to report AFVI occurrence after immune checkpoint inhibitor administration.

## Introduction

Acquired coagulation factor deficiency, caused by coagulation factor inhibitors, has an estimated incidence of one in one million per year. A particularly rare form of this disease is acquired factor V inhibitor (AFVI), which presents with clinical features ranging from asymptomaticity to lethal bleeding. Its possible causes include surgery, medication, malignancy, pregnancy, and autoimmune disorders. We herein report a case of a patient with high-titer AFVI, which developed after administration of nivolumab, an immune checkpoint inhibitor, for recurrent hypopharyngeal cancer. To the best of our knowledge, the present report is the first to describe AFVI occurring after immune checkpoint inhibitor administration. The present study was approved by the Tokyo Metropolitan Tama Medical Center Research Ethics Committee (3-14).

## Case presentation

A 64-year-old male patient presented with a headache and stiff left shoulder of three weeks’ duration. He had no other medical history and was not taking any medication.

An irregular mass was detected in the post-cricoid ring, and a 3-cm lymph node was palpable in the left neck. Based on imaging and histopathological findings, cT1N2cM0 stage IVa hypopharyngeal post-cricoid cancer (squamous cell carcinoma) was diagnosed. Radiotherapy (RT) was administered in five fractions per week for a total of 70 Gy with concurrent, intravenous cisplatin (80 mg/m^2^ on days 1, 22, and 43 of RT) administration every three weeks. Although the local lesion resolved with treatment, lymphadenopathy remained in the left neck, requiring conservative left neck dissection. Although topical bovine and human thrombin preparation were cited in previous reports as causes of AFVI, neither was used during the operation in the present case. Cefazolin sodium was administered four times over two days during the perioperative period to prevent surgical site infection.

Contrast-enhanced CT revealed a recurrence in the anterior neck at postoperative month four, and a tracheotomy was required due to advanced laryngeal edema. Treatment with cisplatin (80 mg/m^2^, day 1), 5-FU (800 mg/m^2^, day 1-5), and cetuximab (400 mg/m^2^, day 1; 250 mg/m^2^, days 8 and 15) was begun but was later switched to nivolumab when the disease showed progression. Tests performed before immune checkpoint inhibitor administration were negative for various autoantibodies. In addition, the result of a coagulation function test at that time was normal. After hospitalization, nivolumab (240 mg flat dose) was administered, but no adverse events were observed, and the patient was discharged the next day. Two weeks later, a blood test at the time of readmission for the second nivolumab course showed prothrombin time (PT) of 96.9 seconds and activated partial thromboplastin time (APTT) of 136.9 seconds, indicating markedly abnormal coagulation function. Platelets were 339 × 10^3^/µL, and D-dimer was 0.9 μg/mL, indicating negativity for disseminated intravascular coagulation (Table 1).

**Table 1:** Result of coagulation tests PT and APTT indicated markedly abnormal coagulation function. PT: prothrombin time; APTT: activated partial thromboplastin time; FDP: fibrin degradation product; TAT: thrombin antithrombin III complex; α2PIC: α2 plasmin inhibitor-plasmin complex

	Day 1 (admission for first nivolumab course)	Day 15	Day 22
PT (10.3–14.4 seconds)	12.2	96.9	98.0
APTT (24.3–36 seconds)	26.0	136.9	151.4
Platelet count (130–400/µL)	160 × 10^3^	339 × 10^3^	
FDP (0–5 µg/mL)			<2.5
TAT (<3 ng/mL)			<1.0
α2PIC (<0.8 µg/mL)			0.5

Table 2 shows that while the activity of all the coagulation factors had decreased, the activity of factor V was below the detection limit (<3%). Measurement of the various coagulation factor inhibitors found that the factor V inhibitor level was as high as 16 Bethesda units (BU)/mL (Table 3). Since the PT and APTT were normal before nivolumab administration, AFVI onset due to nivolumab administration was considered the most likely cause of the coagulation function abnormality observed.

**Table 2:** Coagulation factor activity Factor V activity was below the detection limit. /: unmeasurable

Dilution magnification	1x	2x	3x
Factor Ⅱ activity (%)	5	26	47
Factor V activity (%)	<3.0	/	/
Factor Ⅶ activity (%)	26	50	60
Factor Ⅷ activity (%)	10	60	88
Factor Ⅸ activity (%)	6	52	81
Factor Ⅺ activity (%)	<3.0	37	52
Factor Ⅻ activity (%)	<3.0	26	42

**Table 3:** Coagulation factor inhibitor Factor V inhibitor was as high as 16 BU/mL. BU: Bethesda unit

Factor V inhibitor (BU/mL)	16
Factor Ⅷ inhibitor (BU/mL)	2
Factor Ⅸ inhibitor (BU/mL)	1

In the present case, the failure of the tumor to respond to nivolumab administration prompted the decision to administer palliative treatment. No significant bleeding tendency was observed in the subsequent course, and the AFVI was followed up without treatment. The patient’s condition gradually deteriorated, and he died about a month later.

## Discussion

AFVI is an extremely rare disease first reported in Germany in 1955 [1]. The incidence of AFVI is estimated to be 0.023-0.09 cases per million person-years [2]. Various conditions, such as autoimmune disease, malignancy, infection, antibiotic use, and exposure to topical bovine or human thrombin, can reportedly cause factor V inhibitor formation [1,3]. According to Boland et al., the median age at onset is 74 years (range: 29-90 years), with men being affected more frequently than women (male/female ratio: 2:1) [2]. The presentation of AFVI ranges from asymptomaticity to lethal bleeding. The laboratory findings consist of prolonged PT and APTT [2], low factor V activity, and the presence of factor V inhibitors. In the current case, the PT and APTT were markedly prolonged, factor V was below the detection limit (<3%), and FVI was as high as 16 BU/mL. It should be noted that when the FVI titer is high, the activity of other coagulation factors apparently decreases [4]. In the present case, the activity values for coagulation factors II, VII, VIII, IX, XI, and XII were low, as seen in Table 2, and the increase in the activity of these factors resulting from dilution was thought to be attributable to the effect of FVI (dilution linearity was not observed). Thus, the coagulation factor (II, VII, VIII, IX, XI, and XII) values mentioned above were thought to reflect falsely low levels.

In the present case, factor V activity was thought to have decreased as a symptom of the patient’s condition, but at less than 3%, it was below the detection limit, and dilution linearity was unable to be confirmed. Factor VII and IX inhibitor positivity was considered to be false because the APTT was used as part of the testing method (the Bethesda method) (Table 3). Nakata et al. have reported in detail on this mechanism [4].

The differential diagnosis of AFVI includes congenital factor V deficiency, vitamin K deficiency, anticoagulant use, and antiphospholipid syndrome due to lupus anticoagulant, all of which were ruled out in the present case. Normal coagulation function at the outset denied congenital factor V deficiency. Vitamin K deficiency was also ruled out because the patient had no history of biliary tract disease, coumarin anticoagulant use, malabsorption due to long-term artificial nutrition or intestinal disease, long-term antibiotic use, or liver damage. Although APTT is prolonged in antiphospholipid syndrome, this possibility was ruled out in the present case because of the absence of significant PT prolongation and lupus anticoagulant.

AFVI is treated with hemostasis and immunosuppressive therapy, for which steroids, cyclophosphamide, and rituximab are reportedly effective [5]. Platelet transfusion, fresh frozen plasma, and recombinant activated factor VII are used for hemostasis therapy. However, at present, there is no clear evidence supporting the use of either homeostasis or immunosuppressive therapy.

In recent years, programmed death 1 (PD-1) inhibition with nivolumab has shown better median and two-year overall survival rates and durable responses than systemic therapy in patients with platinum-refractory, recurrent, head and neck squamous cell carcinoma [6]. The antitumor properties of PD-1 inhibition stem from its role as a mediator between T-cells and malignant cells. The binding of PD-1 to PD-L1, one of the two PD-1 receptors on the tumor cell, results in immune checkpoint blockade, inhibiting PD-1 and enhancing T-cell responses to the tumor [7]. However, this enhancement can lead to an autoimmune disorder via failure of self-tolerance when widespread T-cell activation occurs [8]. Various irAE, such as endocrine disorders involving the thyroid gland, pituitary gland, or adrenal gland, gastrointestinal mucosal disorders, interstitial pneumonia, and autoimmune hepatitis, were found to result from the administration of immune checkpoint inhibitors. Therefore, tests for various autoantibodies and thyroid function, chest X-rays, electrocardiograms, and echocardiographic screening tests are required for the safe administration of immune checkpoint inhibitors.

Additionally, in human B-cells where it is also expressed, PD-1 is recruited to the B-cell receptor once it is triggered. PD-1/PD-L1 blockade also increases the activation of B-cells [9]. A previous study demonstrated that antithyroid autoantibodies present at baseline increase after immunotherapy, possibly because of an enhanced humoral response [10].

In their report of a case of acquired hemophilia A, which appeared after nivolumab administration, Gokozan et al. stated that a similar mechanism might play a role in the production of factor VIII inhibitors after immunotherapy in addition to T-cell-related mechanisms [11]. Acquired hemophilia A reportedly appeared after nivolumab administration [11,12], and in the present case, AFVI developed after nivolumab administration. These findings suggested that, in the present patient, factor V deficiency may have resulted from an autoimmune response to factor V caused by nivolumab administration. Because previous reports have described the development of coagulation factor inhibitors following nivolumab administration, coagulation function tests should be performed regularly during nivolumab use.

## Conclusions

We described a case of AFVI with marked prolongation of PT and APTT, which developed in the course of nivolumab therapy for recurrent hypopharyngeal cancer. The time of onset led to the suspicion of AFVI due to nivolumab administration. PD-1/PD-L1 blockade increases the activation of B-cells and T-cells. The same process was thought to be involved in AFVI onset after immunotherapy. To the best of our knowledge, there are no previous reports of AFVI development following nivolumab administration. The presence of coagulation factor inhibitors should be borne in mind as a possible, rare side effect of immune checkpoint inhibitor use.
